# Biotechnological Potential of a Novel Strain of *Fusarium proliferatum*, a Terrestrial Fungus Adapted to Marine Environment

**DOI:** 10.1111/1758-2229.70143

**Published:** 2025-07-04

**Authors:** Antonio Nappo, Michela Salamone, Marco Masi, Michela Morelli, Martina Annunziata, Michele Sonnessa, Alessio Cimmino, Andrea Bosso, Rosanna Culurciello, Ilaria Di Nardo, Elio Pizzo, Maria Costantini, Valerio Zupo, Francesco Aliberti, Marco Guida, Federica Carraturo

**Affiliations:** ^1^ Department of Experimental Medicine University of Campania ‘Luigi Vanvitelli’ Naples Italy; ^2^ Department of Biology University of Naples Federico II Naples Italy; ^3^ Department of Chemical Sciences University of Naples Federico II Naples Italy; ^4^ Bio‐Fab Research Srl Rome Italy; ^5^ Department of Marine Biotechnology Stazione Zoologica Anton Dohrn Napoli Italy; ^6^ HoloBiotics Srl UNINA Spinoff Naples Italy

**Keywords:** antimicrobial, antioxidant, biocompatibility, marine fungi, secondary metabolites

## Abstract

Marine habitats represent hostile environments for the majority of microorganisms. Nonetheless, in the last decades, the study of the microbial diversity of the halophylic environments has reported that fungi constitute a quantitatively relevant component. The research reports the isolation of a novel strain of *Fusarium proliferatum* from seawater, within a monitoring campaign conducted in the South Calabrian coasts (Regione Calabria, Italy): the microorganism presumably adapted from a terrestrial to a marine niche, potentially changing its metabolism in response to the environmental stress. The marine fungus was molecularly characterised preliminarily with Sanger Sequencing, and further employing Whole Genome Sequencing, subsequently cultivated on organic rice to stimulate the production of secondary metabolites. Chemical extraction and purification processes yielded three main compounds identified as 9‐*O*‐methylbostrycoidin (MBC), 9‐*O*‐methylfusarubin (MFR) and 3‐indoleacetic acid (IAA). When tested for their antimicrobial and antioxidant potential, MBC and MFR demonstrated significant activity against *Staphylococcus aureus* and *Listeria monocytogenes* , while IAA exhibited no antimicrobial effect, but highlighted antioxidant properties with the ORAC assay. Additionally, biocompatibility assays on human keratinocyte cells (HaCaT) revealed minimal toxicity of the crude extract and MBC, while IAA displayed dose‐dependent toxicity, opening to considering the purified secondary metabolites for valuable applications in environmental, industrial and pharmaceutical biotechnology.

## Graphical abstract

A novel strain of *Fusarium proliferatum* was isolated from South Calabrian coasts (Italy) and molecularly characterised. Its rice cultures led to the identification of secondary metabolites with antimicrobial activity against *Staphylococcus aureus* and *Listeria monocytogenes* , and with antioxidant properties. Biocompatibility studies have revealed their potential application in the biotechnological field.

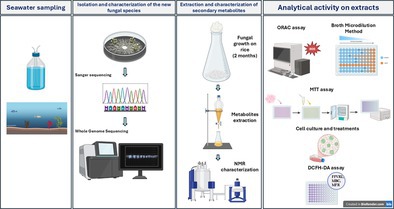

## 1 Introduction

Marine fungi are mainly microscopic forms such as microfungi, yeasts and chytrids, with macroscopic exceptions such as marine lichens, showing a remarkable fungal diversity hidden in a wide range of ecological niches (Richards et al. 2012). These fungi play key ecological roles as parasites (e.g., by infecting phytoplankton), as saprophytes that recycle organic matter and as symbionts with organisms such as algae (Cunliffe 2023). In recent years, several marine fungal species, such as *Penicillium* sp. (Shi et al. 2024), *Aspergillus* sp. (Ha et al. 2024), *Fusarium* sp. (Ameen et al. 2021) and *Alternaria* sp. (Mahmoud et al. 2021) have been studied for biotechnology and represent a promising source of natural bioactive compounds, due to their resistance to extreme conditions and high stability at elevated pH, temperature and salinity. Their secondary metabolites, which include terpenes, steroids, polyketides, peptides, alkaloids and polysaccharides, have antimicrobial, anticancer, antiviral, antioxidant and anti‐inflammatory activities and are therefore useful for medical, pharmacological, agricultural and cosmetic applications. In addition to the production of bioactive compounds, marine fungi are known for their metabolic capacity for bioremediation, which facilitates the degradation of resistant compounds (such as cell walls) and environmental contaminants, including toxic contaminants and microplastics, through enzymes such as catalase, laccase and peroxidase (Zeghal et al. 2021). Environmental monitoring on marine fungi is focused on the research of the two main categories: the ‘obligates’ living exclusively in saline environments, adapted to high salinity and osmotic pressure, and ‘facultatives’ colonising both terrestrial and marine habitats (Gonçalves et al. 2022). Such microorganisms highlight remarkable biochemical adaptations to stress conditions and colonise variable environments from the littoral to the seafloor. Nonetheless, only in recent decades have marine fungi been studied in depth, exploiting culture‐independent approaches and, more recently, next‐generation sequencing (NGS) methods. These techniques allowed us to unveil the mechanisms underlying their unique physiology, proving their capability to genetically adapt to extreme environments (Gonçalves et al. 2022) and uncover new fungal lineages, advancing the understanding of their ecology and evolution (Kumar et al. 2021).

The proposed research started from an environmental hygiene monitoring of marine water and sediments: the campaign allowed the isolation of a wide variety of fungal species, among which, following an initial Sanger sequencing phase, the attention was focused on a strain initially characterised as *Fusarium proliferatum*. *F. proliferatum* represents the asexual (anamorphic) form of the fungus *Gibberella fujikuroi* (sexual or teleomorphic form) (Stanković et al. 2007). It is capable of infecting many plants, including rice, soya, maize, wheat, barley, asparagus, ornamentals, grasses, garlic, figs, onions, palms and pines (Amatulli et al. 2012). *Fusarium* species can also be found within aquatic habitats, including marine and drinking water sources, and some populations appear to be particularly adapted to complex water distribution systems (Steinberg et al. 2015; Zhao et al. 2024). The species is widespread in Asia, Africa, North America and Italy. It is also known to produce secondary metabolites such as bikaverine and fusarin C, and its genome suggests the potential production of several other yet unexplored metabolites. Bioinformatic screening revealed that the *G. fujikuroi* genome encodes 45 key enzymes involved in synthesising secondary metabolites (Yadav et al. 2019). *F. proliferatum* is well known for its pathogenicity to a wide range of cereals, such as wheat, rice, barley, maize, etc. In particular, the fungus is notorious for producing multiple mycotoxins, including fumonisins, moniliformin, fusaric acid, fusarin C and beauvericin (Braun and Wink 2019; Studt et al. 2012). *Fusarium* sp. is also known to produce a large number of other metabolites that have potential industrial applications (e.g., pigments) or because of their biological activities in the medical field. The use of whole cells of *Fusarium* strains as biocatalysts for the conversion and synthesis of aromatic compounds is also increasingly being explored in biotechnology. In this field, the use of microorganisms to produce industrial additives offers advantages over conventional chemical methods. Reactions take place under milder conditions and at lower temperatures, producing less toxic waste and reducing emissions and by‐products (Pessoa et al. 2017).

The novel strain under analysis, submitted on the NCBI database as *F. proliferatum* strain FPI024 Accession number JBJKOO010000000, was isolated from a seawater sample near the mouth of the Crati River along the Calabrian‐Ionian coast (Regione Calabria, Italy). Aiming at assessing the biotechnological potential of the isolate, by evaluating the produced secondary metabolites, a lab‐scale pilot growth system has been tested, exploiting a commercial organic rice ( *Oryza sativa* L.) as a solid matrix under controlled temperature and humidity conditions. Following the extraction process, the biocompatibility with skin cells, antioxidant, antibacterial and antifungal activities of the total extract and the chemically characterised pure metabolites 9‐*O*‐methylbostricoidin, 9‐*O*‐methylfusarubin and 3‐indolacetic acid were assayed.

## 2 Experimental Procedures

### 2.1 Fungal Isolation and Sanger Sequencing

The fungus, obtained during hygiene monitoring and isolated from marine waters collected in the Ionian Sea near the mouth of the river Crati (Regione Calabria, Italy), was cultivated under standard conditions, exhibiting characteristic filamentous growth with white mycelium and slight purplish pigmentation. Fungal DNA extraction was performed using the DNeasy PowerSoil Pro Kit by Qiagen, following the manufacturer's instructions. The DNA sample was amplified with PCR, using a MiniAmp Thermal Cycler, for the fungal characterisation, employing ITS1 forward (5′‐GGA AGT AAA AGT CGT AAC AAG G‐3′, 5′‐TCC GTA GGT GAA CCT GCG G‐3′) and ITS4 reverse (5′‐TCC TCC GCT TAT TGA TAT GC‐3′) primers (Biofab Research, Rome, Italy) complementary to the ITS‐5.8S rDNA region of the fungal 18S rRNA gene (700 bp) (Korabečná et al. 2003). Sequencing reaction was performed by an external service (Biofab Research, Rome, Italy); results were then interpreted using an editing tool, Chromas Lite v. 2.6.6 (Technelysium Pty Ltd., South Brisbane, Australia). The identification of the isolate was conducted using BLASTN ver. 2.2.29 (also referring to GenBank), selecting the identity holding the highest percentage of identity with a 95% cut‐off and a minimum *e*‐value lower than e −460.

### 2.2 Whole Genome Sequencing, Assembly and Annotation of the Novel *Fusarium Proliferatum* Strain

The sample was sequenced on both Illumina MiSeq and Nanopore GridION platforms (Oxford Nanopore Technologies) at the Bio‐Fab Research (IT). The DNA concentration was measured by TapeStation system (Agilent Technologies, California, U.S.), an electrophoresis‐based technique to quantify the concentration based on DNA fragment size. 250 ng were used to prepare sequencing libraries employing standard Illumina DNA Prep Tagmentation and Nextera XT index Kit (Illumina, San Diego, CA, USA) and sequenced on the MiSeq Illumina platform following the manufacturer's instructions. For Nanopore, gDNA library was constructed using 1 μg of DNA by SQK‐LSK109 ligation sequencing kit (Oxford Nanopore Technologies, Oxford, UK) following the manufacturer's instructions. The isolate was sequenced on a GridION device using an R9.4.1 flow cell type for a total of 72 h with the high accuracy (HAC) model (HAC, 450 bps) and a minimum read length of 500 bases. Live base calling was performed using MinKNOW software v24.02.16 and Dorado v7.3.11 for both base calling and barcode demultiplexing in real time.

A total number raw short reads of 17 M paired‐end (PE, 2 × 300) and a total of 10 Gbp (N50 of 8.5 kbp) were generated with Illumina and Nanopore platforms, respectively. Illumina paired‐end raw reads and Oxford Nanopore raw reads files were submitted at NCBI under accession number PRJNA1170920 and Sequence Read Archive (SRA) data as SRR31408079 and SRR31408080, respectively.

Nanopore raw reads were filtered using NanoFilt v2.8.0 (De Coster et al. 2018) to remove reads with a quality score below Q9 or less than 1 kbp in length. Reads passing quality control were used for de novo assembly using Canu v2.2 (Koren et al. 2017) with a specified genome length of 45 Mbp and minimum read length of 1 kbp. The assembled genomes were polished with Pilon (v1.24) (Walker et al. 2014) using Illumina paired‐end reads (300 bp). Finally, the NCBI Foreign Contamination Screen (FCS) was used to identify and remove contaminant sequences in genome assemblies. The sample was assembled into a nuclear genome size of 43,804,295 bp with 12 chromosomes, the longest contig of 6.4 Mbp and the shortest of 0.6 Mbp.

This Whole Genome Shotgun project has been deposited at DDBJ/ENA/GenBank under the accession JBJKOO010000000. The version described in this paper is version JBJKOO010000000. The single contig corresponding to the mitochondrial genome (mtDNA, mitogenome) was assembled with a size of 49,491 bp (Table 1).

**TABLE 1:** Chromosome length and GC content of nuclear genome and mitogenome assemblies.

	Chromosome length	% GC
chr1	6,405,240	0.4876
chr2	4,821,243	0.4856
chr3	4,876,672	0.4866
chr4	4,404,685	0.4817
chr5	4,372,117	0.4866
chr6	4,105,736	0.4849
chr7	3,269,181	0.4848
chr8	3,145,555	0.4777
chr9	2,961,924	0.4829
chr10	2,543,996	0.4748
chr11	2,279,435	0.4787
chr12	618,511	0.4342
Mit	49,491	0.3185

**FIGURE 1:**
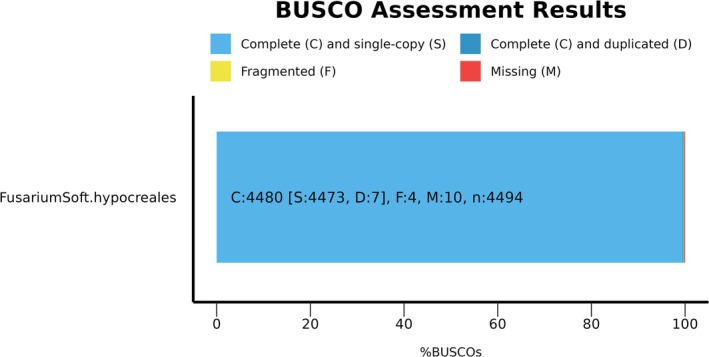
BUSCO assessment results to Hypocreales lineage with 99.7% genome completeness for 4494 number of BUSCOs.

Genome completeness was then predicted using BUSCO v5.7.1 coupled with dataset odb10 version and auto‐lineage eukaryotic (Figure 1) (Seppey et al. 2019).

RepeatModeler v2.0.3 and RepeatMasker v4.1.3‐p1 run with rmblastn version 2.14.1+ were used to identify repetitive content. Up to 10 different classes of repeats identified were soft‐masked (1,030,652 bp, 2.35%). Total interspersed repeats as retroelements (SINEs, LINEs, LTR elements), DNA transposons, rolling‐circles and also unclassified account ~1.63%. Furthermore, small RNA (0.06%), simple repeats (0.56%) and low complexity (0.08%). Augustus v3.5.0 (Stanke et al. 2008) was used for de novo protein prediction. Ab initio gene prediction detects 13,930 complete protein‐coding genes. In the near future, to improve ab initio annotation will use evidence from RNA‐seq data.

### 2.3 Fungal Isolates and Cultures

The fungal isolate FPI024, identified by Sanger sequencing and WGS as a putative member of the species *F. proliferatum*, was subsequently reactivated from cryopreserved glycerol stocks by aerobic cultivation at 25°C in Czapek‐Dox broth (Thermo Scientific, Massachusetts, USA) under agitation conditions. This was followed by solid culture. The solid matrix was composed of organic rice with the lowest possible level of potential contaminants, which was autoclaved at 121°C for 50 min. 300 g of rice, to which 30% distilled water was added, was placed in an Erlenmeyer flask and subsequently sealed with cotton and foil. The rice was then incubated in a thermostat at 25°C for 2 months. This allowed the fungus to colonise the inside of the caryopses. At the end of the growth period, the culture was dried in an oven at about 40°C for 24 h.

### 2.4 Chemical Characterisation

#### 2.4.1 General Experimental Conditions

Column chromatography (CC) was performed on silica gel (Merck, Kieselgel 60, 0.063–0.200 mm, Merck, Darmstadt, Germany). Thin Layer Chromatography (TLC) was performed on silica gel on direct (Kieselgel 60, F_254_, 0.25 or 0.50 mm) and reverse phase (Whatman, KC18, F_254_, 0.20 mm) (Merck, Darmstadt, Germany) plates. The spots were visualised by exposure to UV radiation (254 nm) or by spraying first with 10% H_2_SO_4_ in methanol, followed by heating at 110°C for 10 min. ^1^H‐NMR spectra were recorded at 400 MHz on a Bruker (Karlsruhe, Germany) Anova Advance spectrometer. The spectra were recorded in deuterated solvents, which were also used as internal standards. Electrospray Ionisation Mass Spectra (ESI‐MS) were performed using the LC/MS TOF system AGILENT 6230B (Agilent Technologies, Milan, Italy), HPLC 1260 Infinity. Sigma‐Aldrich Co. (St. Louis, MO, USA) supplied all the reagents and the solvents.

#### 2.4.2 Secondary Metabolites Extraction, Purification and Identification

The solid culture was placed in a blender with a 600 mL solution of MeOH/H_2_O (1:1, *v/v*) and macerated at room temperature. After 24 h, the mixture was centrifuged at 7000 rpm for 20 min to promote separation between the solid residue and the hydroalcoholic solution. The supernatant was subjected to liquid–liquid extraction using CH_2_Cl_2_ (2 × 300 mL) as the organic solvent. The solvent was then removed under reduced pressure, yielding a residual crude extract of 319.2 mg. This latter was used for biological assays, and 280.0 mg were purified by CC eluted with a mixture of CHCl_3_/MeOH (9.5:0.5, *v/v*), followed by a final elution with MeOH. Six homogeneous fractions have been collected: F1 (65.4 mg), F2 (20.9 mg), F3 (44.8 mg), F4 (22.6 mg), F5 (30.8 mg), F6 (38.4 mg). The residue of F2 was further purified using CH_2_Cl_2_/MeOH (8.5/0.5, *v/v*) as eluent, yielding a pure compound which was identified as 9‐*O*‐methylbostrycoidin (MBC, 6.1 mg). The residue of F3 was further purified using the same eluent employed for F2 purification, yielding a further amount of MBC (2.3 mg for a total of 8.4 mg) and another pure compound, which was identified as 9‐*O*‐methyl fusarubin (MFR, 30.2 mg). The residue of F5 was further purified by reverse phase TLC eluted with a mixture of MeCN/H_2_O (1:1, *v/v*), yielding a pure compound which was identified as 3‐indoleacetic acid (IAA, 6.3 mg).

9‐*O*‐Methylbostrycoidin (MBC): Its ^1^H NMR data were in agreement with those already reported in the literature (Masi et al. 2018); ESI‐MS (+) *m/z* 300 [M + H]^+^.

9‐*O*‐Methylfusarubin (MFR): Its ^1^H NMR data were in agreement with those already reported in literature (Masi et al. 2018); ESI MS (+) *m/z* 321 [M + H]^+^.

3‐Indoleacetic acid (IAA): Its ^1^H‐NMR data were in agreement with those already reported in literature (Cimmino et al. 2006); ESI‐MS (−) *m/z* 174 [M−H]^−^.

### 2.5 Antimicrobial Activity Assays

#### 2.5.1 Kirby‐Bauer Disk Diffusion Method

The Kirby‐Bauer a was used for preliminary evaluation of potential antimicrobial activity against target microorganisms: *Staphylococcus aureus* ATCC 25923, *Listeria monocytogenes* ATCC 19115, *Escherichia coli* ATCC 8739, *Salmonella enterica* serovar Typhimurium ATCC 14028, *Enterococcus hirae* ATCC 10541, *Campylobacter jejuni* ATCC 33291, *Pseudomonas aeruginosa* ATCC 27853 and *Candida albicans* ATCC16053. An inoculum of 10^7^ CFU mL^−1^ was prepared, and 1 mL of the inoculum was plated on Mueller‐Hinton agar. Oxoid Antimicrobial Susceptibility Test Discs were impregnated with 10 μL of the test substances at different concentrations dissolved in dimethylsulphoxide (DMSO). Specifically, 1 mg of the crude extract was tested, whereas 0.1 mg, 0.05 mg and 0.01 mg were tested for the pure compounds. For negative and positive controls, discs were soaked with 10 μL DMSO and 10 μL hydrogen peroxide disinfectant. All experiments were performed in triplicate. The plates were incubated at 37°C for 24 h for the bacterial species and at 22°C for 96 h for the fungal species, and the diameter of the inhibition zone was measured. The compounds' potential antimicrobial activity is expressed in millimetres (mm).

#### 2.5.2 Minimum Inhibitory Concentration (MIC)

Minimum inhibitory concentration (MIC) determination of crude extract and pure molecules was performed by broth microdilution method according to Clinical and Laboratory Standards Institute (CLSI) (Zimmer 2024). The assays were performed in 96‐well multiwell plates (ThermoScientific). The final volume was 200 μL. To obtain plate concentrations of 100–0.39 μg mL^−1^, the compounds to be tested were diluted in DMSO. In this way, the compounds were brought into contact with the inocula of 2 × 10^6^ CFU mL^−1^ of the following target micro‐organisms: *Staphylococcus aureus* ATCC 25,923, *Listeria monocytogenes* ATCC 19,115, *Escherichia coli* ATCC 8739, *Salmonella enterica* serovar Typhimurium ATCC 14,028, *Enterococcus hirae* ATCC 10,541, *Campylobacter jejuni* ATCC 33,291, *Pseudomonas aeruginosa* ATCC 27,853 and *Candida albicans* ATCC 16,053.

A commercially available peroxide‐based disinfectant and a solvent for the substance (DMSO) were used as the positive and negative controls, respectively. The microplate was incubated for 24 h at 37°C with continuous shaking (600 rpm). The microplate was read on a Thermo Scientific Varioskan LUX multimode microplate reader. Turbidity readings were taken at hourly intervals from 0 to 24 h, for a total of 25 readings, at a wavelength of 560 nm. The test was performed in triplicate. The following formula was used to determine the percentage inhibition of microbial growth:

$$\begin{array}{c} \%\text{Growth inhibition}=100\;\times \left(1-\frac{\mathrm{Abs}\;560\;\mathrm{nm of test sample}}{\mathrm{Abs}\;560\;\mathrm{nm of CTR}}\right) \end{array}$$

An ANOVA analysis was conducted (GraphPad Prism Software version 9 for Windows, GraphPad Software, La Jolla, California, USA, www.graphpad.com) to identify the statistical significance between the sample and positive control. *p* < 0.05 were considered statistically significant and are indicated in the graphs as *p < 0.05, **p < 0.01, ***p < 0.001 and ****p < 0.0001.

### 2.6 Antioxidant Activity Assay (ORAC Assay)

The antioxidant potential was evaluated using the ORAC (oxygen radical absorbance capacity) method. Typically, Trolox equivalents (TE) are used to express the antioxidant capacity of the samples tested. The test was performed in 96‐well plates (Thermo Scientific Nunc F96 MicroWell Black Polystyrene Plate‐Thermo Scientific 237,108). The final volume was 200 μL. The following reagents were used: Fluorescein sodium salt (Sigma‐Aldrich F6377); Trolox (6‐hydroxy‐2,5,7,8‐tetramethylchroman‐2‐carboxylic acid) (Sigma‐Aldrich 238,813); AAPH (2,2′‐azobis (2‐methylpropionamidine) dihydrochloride) (Sigma‐Aldrich 440,914); and phosphate (buffer‐10 mM, pH 7. 4). The protocol indicated by the manufacturer Thermo Scientific ‘*Determination of Antioxidant Capacity on the Thermo Scientific Varioskan LUX Multimode Reader*’ was then followed.

### 2.7 Cell Culture and Treatment

Immortalised human keratinocytes (HaCaT cells) were cultured in Dulbecco's modified Eagle medium (DMEM) supplemented with 10% foetal bovine serum (FBS), 1 mM L‐ glutamine, 1% v/v penicillin/streptomycin solution (100 Unit/mL) and grown at 37°C in 5% CO_2_ and treated for biocompatibility and antioxidant assay as in (Culurciello et al. 2022). Different doses of crude extract, MBC, MFR and IAA were administered for 6, 24 and 48 h to perform biocompatibility assays. Instead, oxidative stress was induced with 100 μM H_2_O_2_ (Merck KGaA, Darmstadt, Germany) for 24 h. For the following experiments, treatments were carried out by using increasing amounts of FFVR1.

### 2.8 Cell Viability Assay on HaCaT Cells

The cytotoxic effects of extracts on HaCaT cells were determined by performing an MTT assay designed to be used for the spectrophotometric quantification of cell proliferation. Briefly, 5 × 10^3^ cells were seeded into a 96‐well plate and incubated at 37°C in the presence of 5% CO_2_. Cells were treated with increasing doses of extracts at a final concentration ranging from 6.25 to 400 μg mL^−1^. After 6, 24 and 48 h of incubation at 37°C, the media were removed, and 100 μL of tetrazolium MTT diluted at 0.5 mg mL^−1^ in Dulbecco's modified Eagle's medium (DMEM) purchased from Lonza (Basel, Switzerland) without red phenol was added. After 4 h of incubation at 37°C, the resulting insoluble formazan salts were solubilised in 0.04 M HCl in anhydrous isopropanol and quantified by measuring the absorbance at *λ* = 570 nm, using an automatic plate reader spectrophotometer (Synergy H4, USA). Cell survival was expressed as the mean of the percentage value compared to the control.

### 2.9 DCFH‐DA Assay

The ROS quantification assay was carried out by using the DCFH‐DA (2′,7′‐dichlorofluorescin diacetate). 2 × 10^4^ cells were seeded into a 96‐well plate and incubated at 37°C in a 5% CO_2_ atmosphere overnight. Then, cells were washed in PBS 1×, opportunistically treated and incubated with 20 μM DCFH‐DA at 37°C for 40 min. After the incubation time, the fluorescence of the cells from each well was measured and recorded in the excitation/emission wavelengths of 485–532 nm, by means of a multimode microplate reader (Synergy H4).

### 2.10 Statistical Analyses

Statistical analyses were carried out by using GraphPad Prism. Values are reported as the means ± SD of biological replicates (*p < 0.05, **p < 0.01, ***p < 0.001 or ****p < 0.0001) compared to the respective controls (one‐way ANOVA, followed by Bonferroni's posttest).

## 3 Results

### 3.1 Fungal Isolation, Sanger Sequencing and Whole Genome Sequencing

**FIGURE 2:**
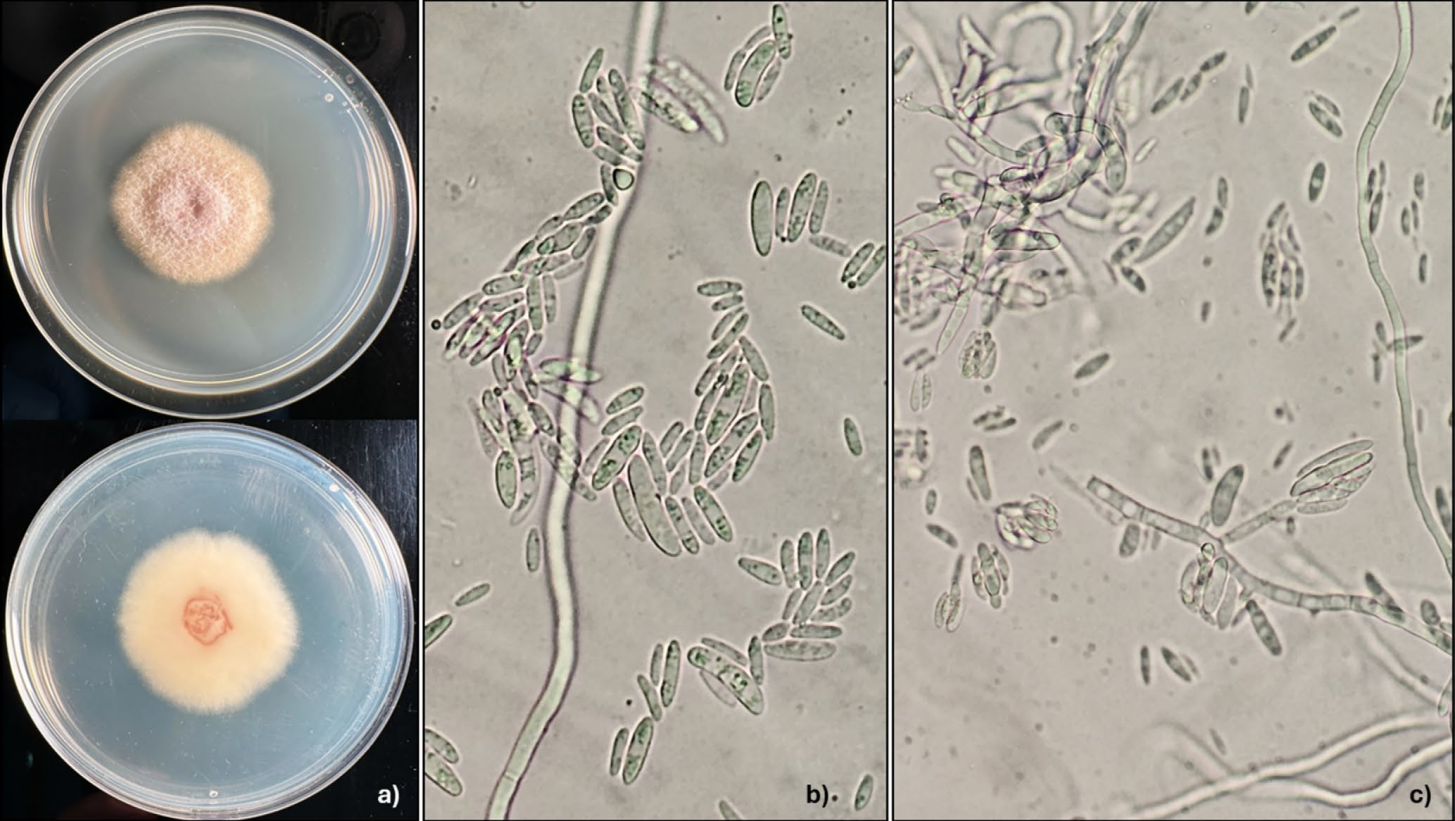
Morphological features of FPI024 (a) Colony on PDA plate after days of incubation at 25°C: Top view (top) and reverse (bottom); and (b, c) microscopic images observed under light microscopy (40× magnification) showing septate hyphae and numerous ellipsoidal to fusiform conidia, arranged in clusters and borne on branched conidiophores.

The isolated novel strain has been morphologically characterised: the colonies are consistently similar to the *Fusarium proliferatum* characteristic morphology (Punja 2020) (the plate is available in Figure 2).

The preliminary molecular characterisation of the novel strain, conducted through Sanger sequencing, revealed a 97.40% identity with *F. proliferatum*, as shown in Table 2.

**TABLE 2:** Results of Sanger sequencing for the first molecular classification of the isolated fungus.

Isolated microorganism	Total score	Query cover	e‐value	%Identity	Accession no.
*Fusarium proliferatum* isolate 4	654	100%	0.0	97.40%	OR10192

**FIGURE 3:**
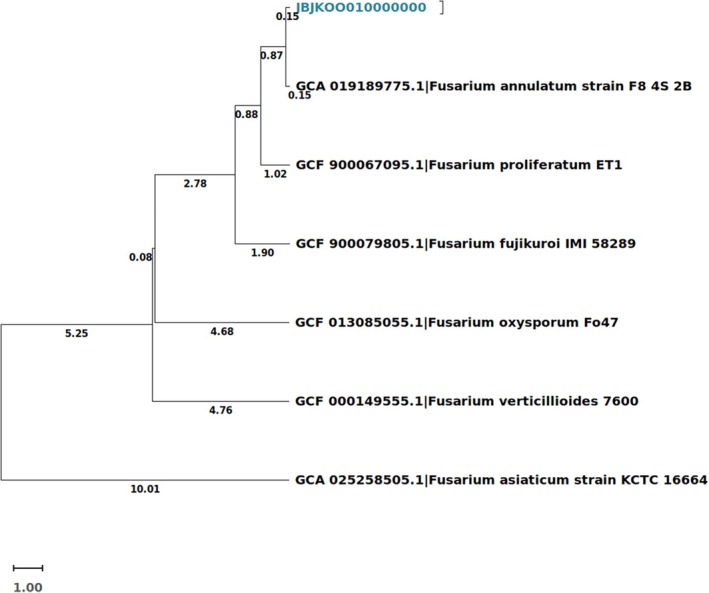
Phylogenomic tree of six *Fusarium* assemblies and JBJKOO010000000 by Hierarchical clustering of taxa with AAI values by UPGMA method.

In order to confirm the lineage, a phylogenomic analysis was performed: our assembly was compared with six *Fusarium* assemblies chose based both on BLAST results (per cent identity > 97%) of conserved genes like calmodulin (*cmd*A), RNA polymerase largest subunit (*rpb*1), RNA polymerase second largest subunit (*rpb*2), partial sequences of the translation elongation factor 1‐alpha (*tef*1), beta‐tubulin (*tub*2) (Gomez‐Chavarria et al. 2024; Lombard et al. 2019) and the matched *Fusarium* complete assemblies. The average amino acid identity (AAI) score was calculated using MMseqs2 v17.b804f from EzAAI v1.2.3 (Kim et al. 2021) on extracts profile database by Prodigal V2.6.3 of *Fusarium proliferatum* starin ET1 (GCF_900067095.1), *Fusarium fujikuroi* IMI 58289 (GCF_900079805.1), *Fusarium oxysporum* Fo47 (GCF_013085055.1), *Fusarium verticillioides* 7600 (GCF_000149555.1), *Fusarium annulatum* strain F8_4S_2B (GCA_019189775.1), *Fusarium asiaticum* strain KCTC 16664 (GCA_025258505.1) and JBJKOO010000000. JBJKOO010000000 resolved in the same node with isolate named ‘*Fusarium annulatum* strain F8 4S 2B’. Our strain and *Fusarium annulatum* strain F8 4S 2B are delimited in the *Fusarium fujikuroi* species complex (FFSC), together with *Fusarium proliferatum* strain ET1. Noteworthy, Nelson et al. (1983) mentioned that *F. annulatum* is essentially a *F. proliferatum* with strongly curved sporodochial conidia (Figure 3) (Yilmaz et al. 2021).

*Fusarium proliferatum* strain FPI024 Accession number JBJKOO010000000 shares a high 18S rRNA similarity to other species of the *Fusarium proliferatum* genus. Genome analysis revealed the presence of genes associated with the fusarubin cluster, indoleacetamide hydrolase and tryptophan‐2‐monooxygenase, which are involved in the synthesis of secondary metabolites with antimicrobial, antiviral, antitumor, antioxidant, antiparasitic and immunomodulatory activities (Zeghal et al. 2021).

### 3.2 Extraction and Purification of Secondary Metabolites

**FIGURE 4:**
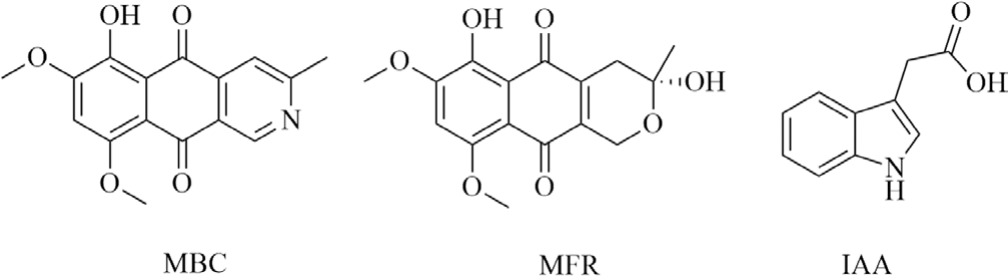
Chemical structures of the pure metabolites: 9‐*O*‐methylbostrycoidin (MBC), 9‐*O*‐methylfusarubin (MFR) and 3‐indoleacetic acid (IAA).

The solid culture was extracted with a hydroalcoholic solution and then with CH_2_Cl_2_, yielding a crude organic extract which was purified by CC and TLC, obtaining three pure compounds. The pure compounds were identified by comparing their spectroscopic data (essentially ^1^H NMR, Figures S1–S3) with those reported in the literature as 9‐*O*‐methylbostrycoidin (MBC) (Masi et al. 2018), 9‐*O*‐methylfusarubin (MFR) (Masi et al. 2018) and 3‐indoleacetic acid (IAA) (Cimmino et al. 2006) (Figure 4).

Their identification was confirmed by the data obtained from the ESI‐MS spectra recorded in positive or negative mode (Figures S4–S6) that showed the protonated pseudomolecular ion [M + H]^+^ peaks at *m*/*z* 300 and 321 for MBC and MFR, respectively, and the deprotonated pseudomolecular ion [M‐H]^−^ peak at *m*/*z* 174 for IAA.

### 3.3 Antimicrobial Activity

#### 3.3.1 Kirby‐Bauer Disk Diffusion Method

The antibacterial and antifungal activity of the crude extract and isolated pure compounds was evaluated by the Kirby‐Bauer disc diffusion method, whose zone of inhibition values are reported in Table 3. The diameters of the zone of inhibition induced by a concentration of 1 mg/disk of the crude extract were 20.0 + 0.2 mm for *S. aureus* ATCC 25923, 10.0 + 0.15 mm for *L. monocytogenes* ATCC 19115, 10.0 + 0.12 mm for *E. hirae* ATCC 10541 and 8.0 + 0.1 mm for *C. albicans* ATCC 16053. For *E. coli* ATCC 8739, *Salmonella enterica* serovar Typhimurium ATCC 14028, *C. jejuni* ATCC 33291 and *P. aeruginosa* ATCC 27853, no growth inhibition was observed around the disks soaked with the tested extract. The diameter of the zone of inhibition induced by 100 μg/disc of 9‐*O*‐methyl bostrycoidin was 9.0 + 0.1 mm for *S. aureus* ATCC 25923, whereas no halo was observed at 50 and 10 μg. Similarly, no inhibition halos were recorded for the other target microorganisms. For 9‐*O*‐methyl fusarubin, an inhibition halo of 8.0 + 0.1 mm was measured at 100 μg, while at 50 μg, a halo of 7.0 + 0.2 mm was measured for *S. aureus* ATCC 25923. No inhibition halo was recorded at 10 μg. For 3‐indoleacetic acid, no inhibition halo was recorded at any level tested. No halo of inhibition was observed when the microorganisms were treated with DMSO (CTR−), the solvent of the crude extract.

**TABLE 3:** Antimicrobial activity of crude extract and isolated compounds assessed using the Kirby‐Bauer disk diffusion method. Results are reported as inhibition zone diameters (mm) ± standard deviation (SD) against tested bacterial strains (n.a. = not active).

Kirby‐Bauer disk diffusion method	Inhibition zone (mm)											
	Crude extract	9‐*O*‐Methyl bostrycoidin (MBC)			9‐*O*‐Methyl fusarubin (MFR)			3‐Indoleacetic (IAA)			CTR^+^	CTR−
Target microorganism	1 mg	100 μg	50 μg	10 μg	100 μg	50 μg	10 μg	100 μg	50 μg	10 μg		
*Staphylococcus. aureus* ATCC 25923	20 ± 0.2	9 ± 0.1	0	0	8 ± 0.1	7 ± 0.2	0	0	0	0	31 ± 0.1	0
*Listeria monocytogenes* ATCC 19115	10 ± 0.15	0	0	0	0	0	0	0	0	0	17 ± 0.2	0
*Escherichia coli* ATCC 8739	0	0	0	0	0	0	0	0	0	0	20 ± 0.3	0
*Enterococcus hirae* ATCC 10541	10 ± 0.12	0	0	0	0	0	0	0	0	0	15 ± 0.1	0
*Salmonella enterica serovar Typhimurium* ATCC 14028	0	0	0	0	0	0	0	0	0	0	20 ± 0.3	0
*Campylobacter jejuni* ATCC 33291	0	0	0	0	0	0	0	0	0	0	19 ± 0.1	0
*Pseudomonas aeruginosa* ATCC 27853	0	0	0	0	0	0	0	0	0	0	20 ± 0.2	0
*Candida albicans* ATCC 16053	8 ± 0.1	0	0	0	0	0	0	0	0	0	11 ± 0.3	0

#### 3.3.2 Broth Microdilution Method

**FIGURE 5:**
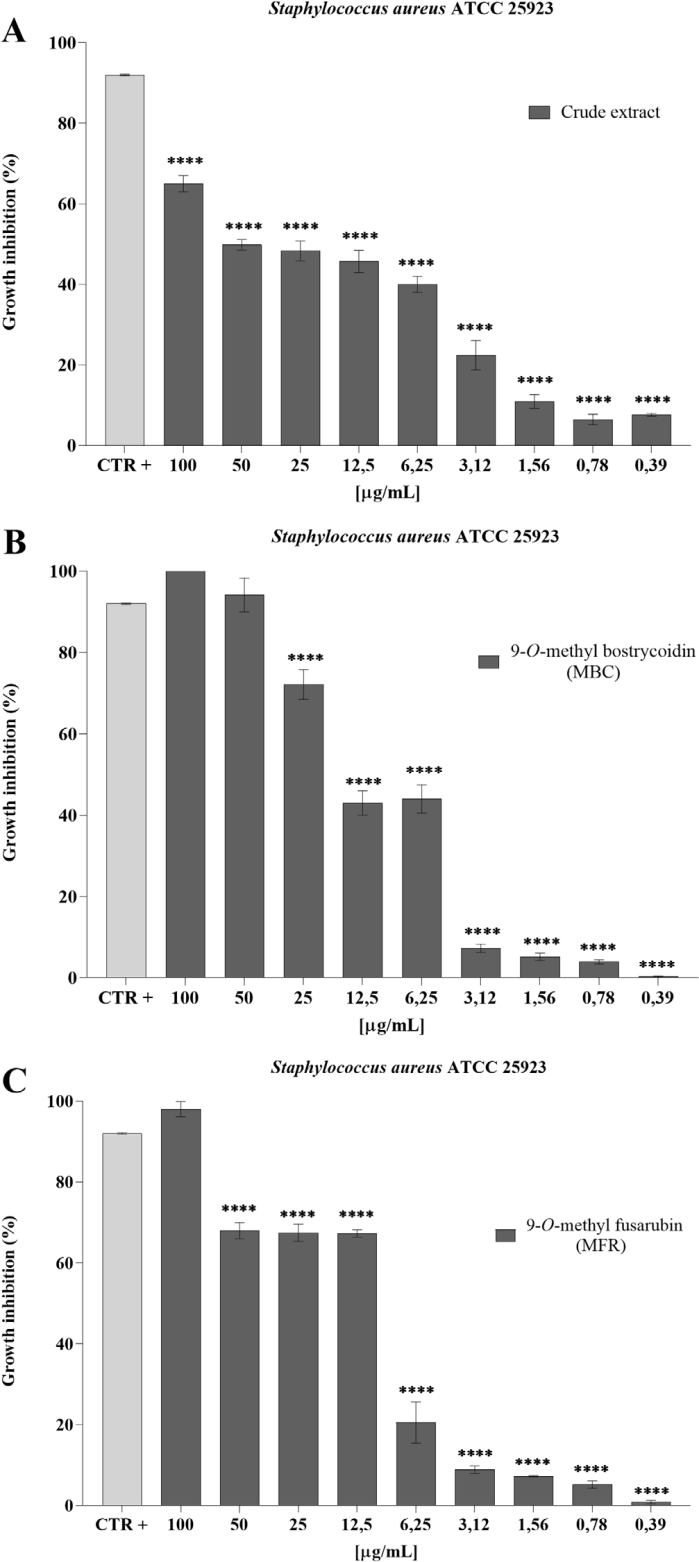
Antibacterial activity of crude extract (A), MBC (B) and MFR (C) against *Staphylococcus aureus* ATCC 25923, ****: *p* < 0.0001.

The crude extract produced a dose‐dependent inhibition of growth of *Staphylococcus aureus* ATCC 25923, with the highest effect (approximately 60%) recorded at a concentration of 100 μg/mL. 9‐*O*‐methyl bostrycoidin (MBC) resulted in 100% inhibition at a concentration of 100 μg mL^−1^ and greater than 90% inhibition at a concentration of 50 μg mL^−1^. The MIC50 value is 25 μg mL^−1^. Concentrations of 12.5 and 6.25 μg mL^−1^ showed growth inhibition of approximately 40%. Finally, no inhibitory effect on target microorganism viability was observed at lower concentrations. With regard to the antibacterial activity of 9‐*O*‐methyl fusarubin (MFR), the growth inhibition of 98.01% was observed at the highest concentration tested. A growth inhibition of approximately 67% was observed at concentrations between 50 and 12.5 μg mL^−1^. The MIC50 value is 12.5 μg mL^−1^. A nonsignificant inhibition of microbial growth was observed at decreasing concentrations (Figure 5).

**FIGURE 6:**
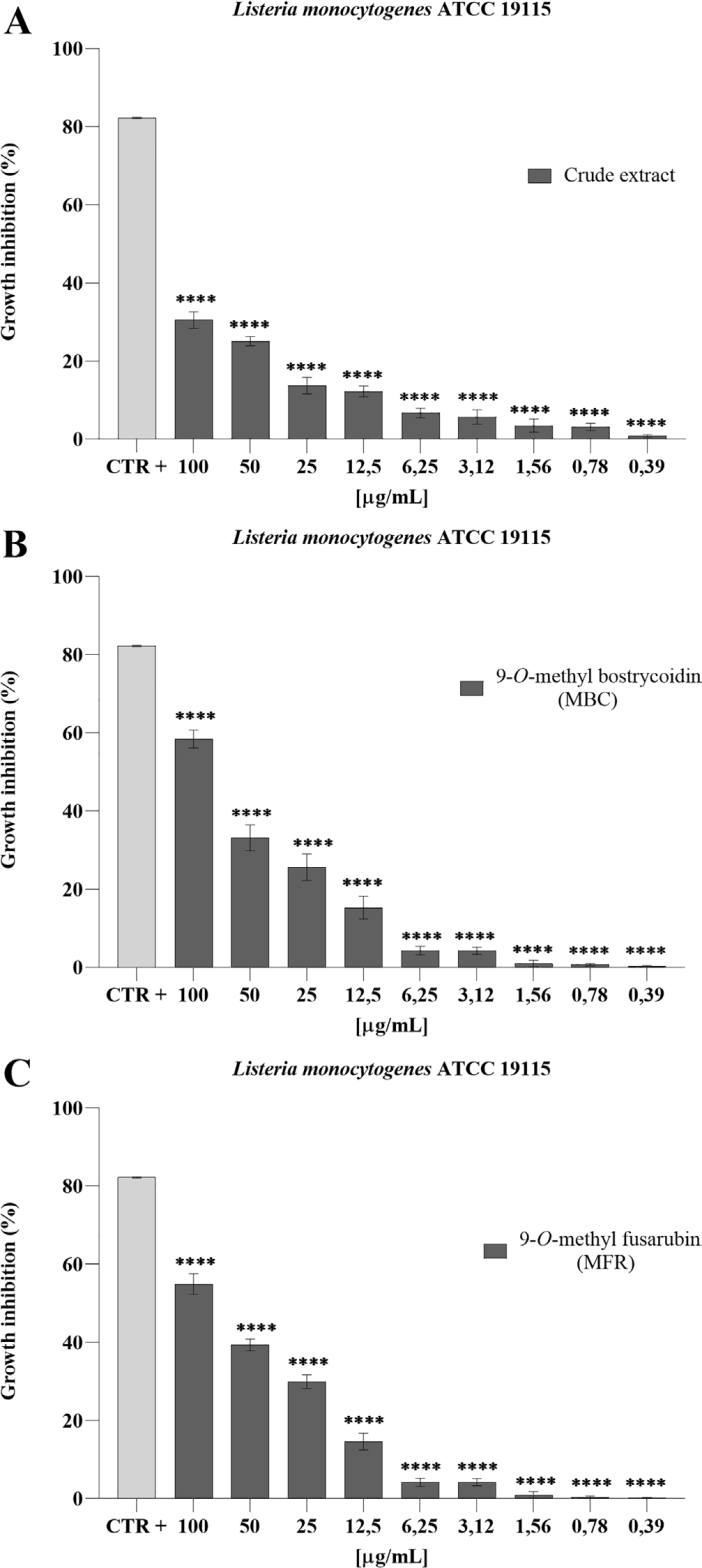
Antibacterial activity of crude extract (A), MBC (B) and MFR (C) against *Listeria monocytogenes* ATCC 19115, ****: *p* < 0.0001.

Regarding the antibacterial activity of the crude extract against *Listeria monocytogenes* ATCC 19115, no significant change in the growth of this microorganism was observed even at the highest concentration tested. In fact, at a concentration of 100 μg mL^−1^, the maximum growth inhibition was 30%. For the two pure compounds 9‐*O*‐methyl bostrycoidin and 9‐*O*‐methyl fusarubin, a percentage of growth inhibition was observed that decreased with decreasing concentration tested and at a concentration of 100 μg mL^−1^ showed a 59.91% inhibition of the target micro‐organism, whereas for 9‐*O*‐methyl fusarubin the inhibition was 50% at the highest concentration tested (Figure 6).

**FIGURE 7:**
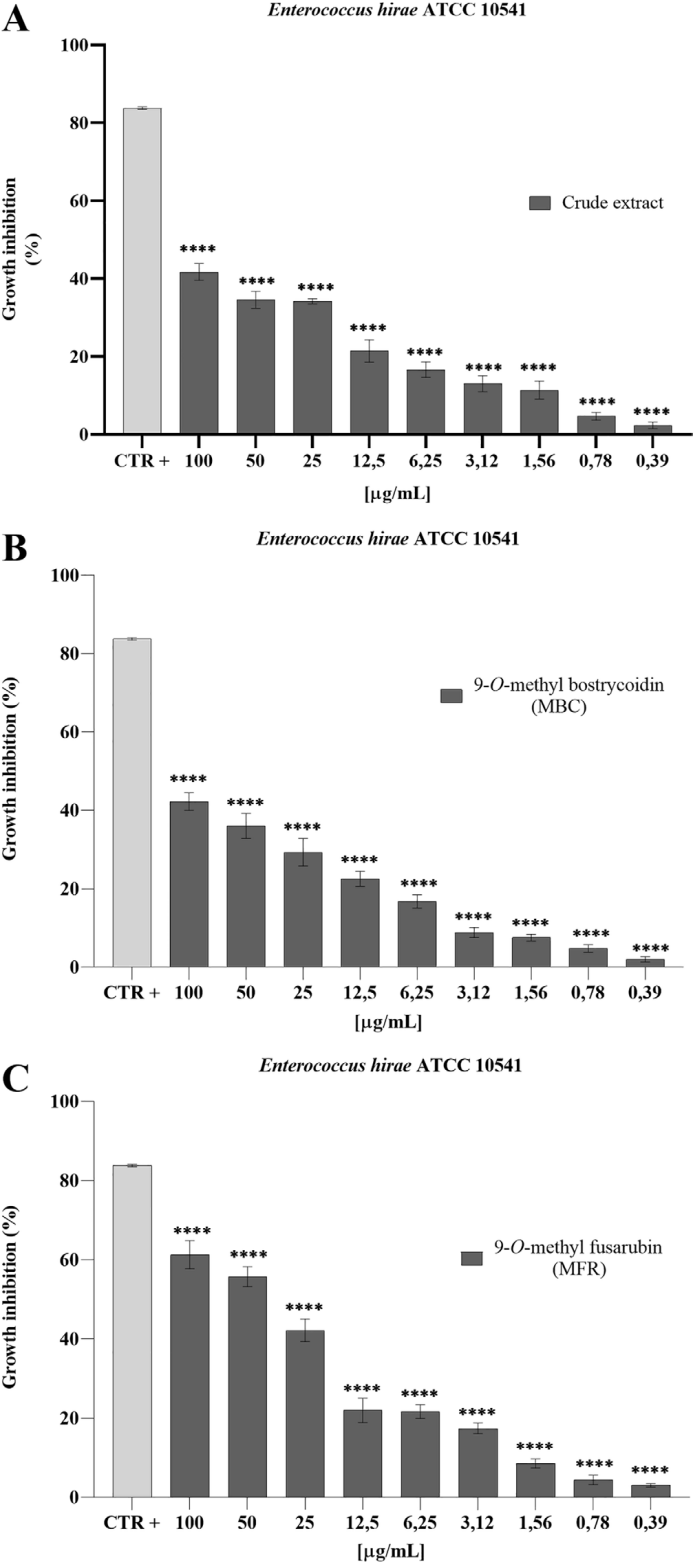
Antibacterial activity of crude extract (A), MBC (B) and MFR (C) against *E. hirae* ATCC 10541, ****: *p* < 0.0001.

**FIGURE 8:**
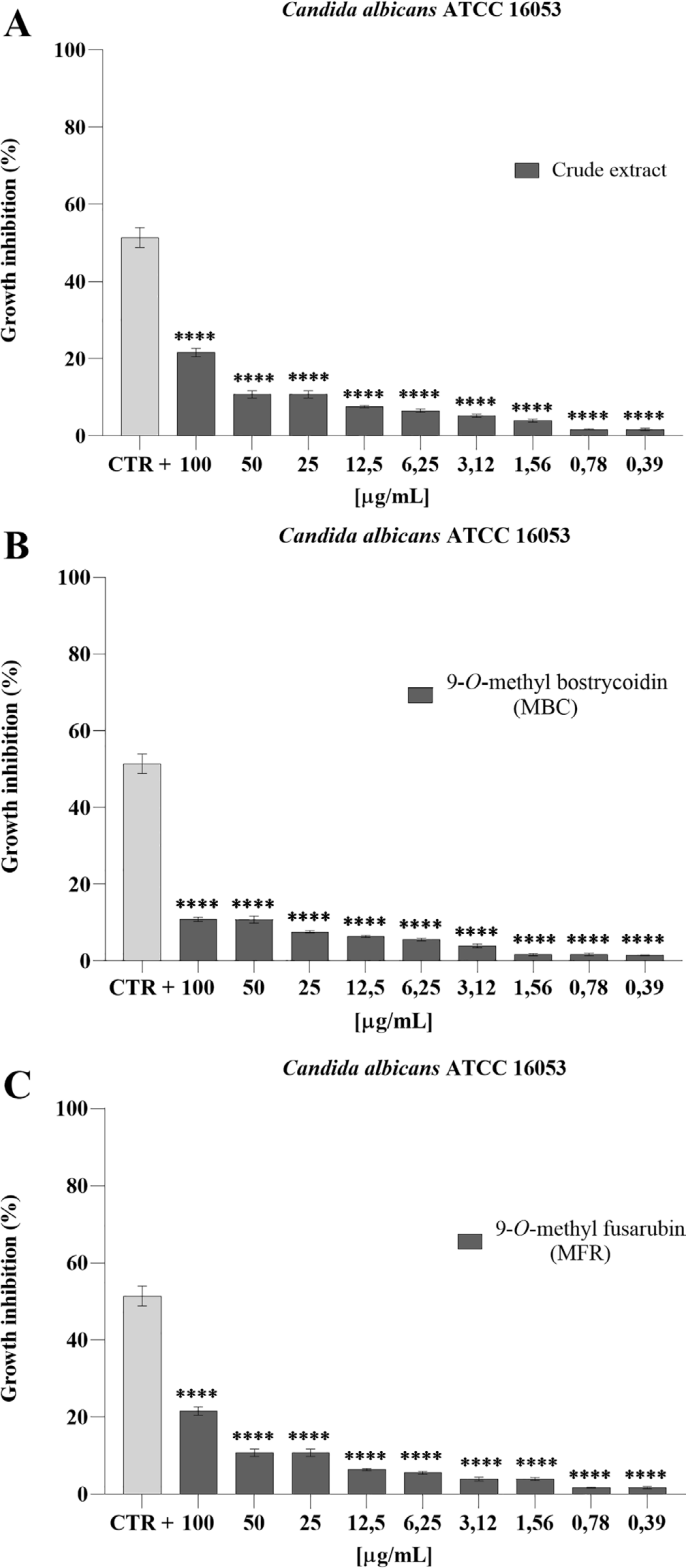
Antifungal activity of crude extract (A), MBC (B) and MFR (C) against *C. albicans* ATCC 16053, ****: *p* < 0.0001.

The effect of the crude extract against *Enterococcus hirae* ATCC 10541 was not significant in terms of growth inhibition, even at the highest concentration tested (100 μg mL^−1^). When exposed to 9‐*O*‐methyl bostricoidin and 9‐*O*‐methyl fusarubin, growth inhibition, expressed in %, decreased as the concentration tested decreased. The maximum inhibition recorded was 42.22% and 60.0% at a concentration of 100 μg mL^−1^ for MBC and MFR, respectively (Figure 7). The effect of the crude extract on *Candida albicans* ATCC 16053 showed no significant inhibition of the test microorganism. A maximum percentage (about 20%) of growth was recorded at the highest concentration tested (100 μg mL^−1^) with respect to 9‐*O*‐methyl fusarubin (Figure 8). All data recorded at the different concentrations were statistically significant compared to CTR+ (*p* < 0.0001).

### 3.4 Antioxidant Activity (ORAC Assay)

The results obtained from the ORAC assay showed that both 9‐*O*‐methyl bostricoidin and 9‐*O*‐methyl fusarubin did not exhibit antioxidant activity. Conversely, 3‐indoleacetic acid, which had shown no potential antibacterial and antifungal activity, showed potential antioxidant activity. Table 4 shows the antioxidant activity of 3‐indoleacetic acid (IAA) measured as Trolox Equivalent Antioxidant Capacity (TEAC) at different concentrations (μg mL^−1^). TEAC values indicate the ability of the compound to neutralise free radicals, with higher values reflecting greater antioxidant activity. The antioxidant capacity of IAA peaks at concentrations around 25 μg mL^−1^, where it achieves a TEAC of 360.24. This value represents the highest antioxidant potential observed across the concentrations tested. The TEAC values show a general decrease as the concentration decreases from 6.25 to 0.39 μg mL^−1^, indicating that the antioxidant efficacy decreases at lower concentrations. At 0.39 μg mL^−1^, the TEAC value drops significantly to 144.06, suggesting a reduced ability to scavenge free radicals at this low concentration.

**TABLE 4:** ORAC assay results for 3‐indoleacetic acid, expressed as μM Trolox equivalents.

3‐Indoleacetic acid (IAA)	
[μg/mL]	Trolox equivalent [μM] ± SD
25	360,24 ± 2,64
12,5	358,69 ± 2,28
6,25	348,01 ± 3,03
3,12	336,82 ± 0,22
1,56	274,85 ± 5,24
0,78	183,97 ± 3,64
0,39	144,06 ± 0,87

### 3.5 Effects of the Crude Extract on Human Keratinocytes HaCaT Cells

**FIGURE 9:**
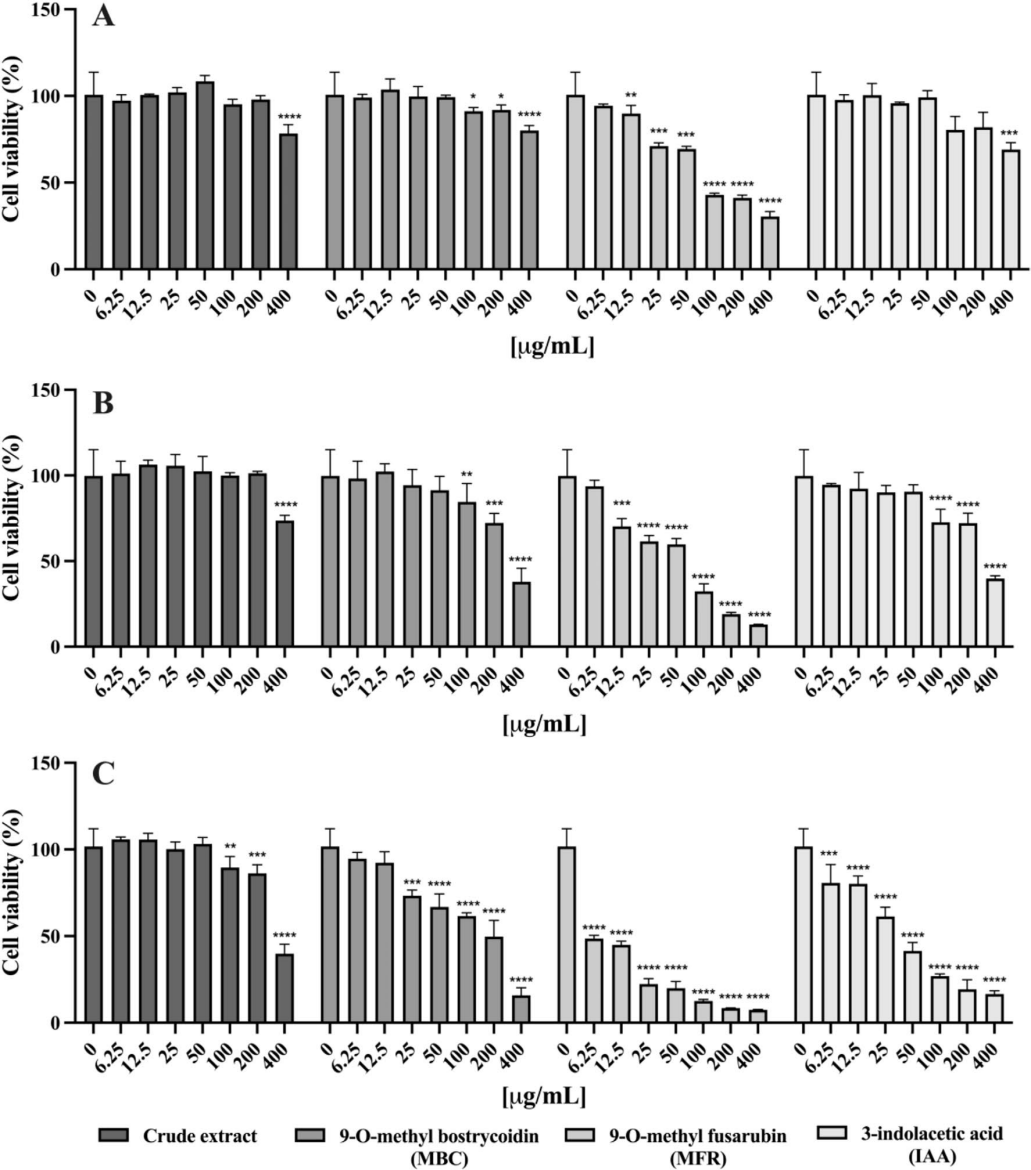
Cell viability on HaCaT cells treated with crude extract, MBC, MFR and IAA. Increasing concentrations of extracts (from 6.25 to 400 μg mL^−1^) were administrated to HaCaT cells for 6 (A), 24 (B) and 48 h (C). Cell viability was determined by the MTT assay. Statistical analysis was carried out by GraphPad Prism using Student's *t*‐test (*p < 0.05, **p < 0.01, ***p < 0.001, ****p < 0.0001).

The antioxidant activity of the extract highlighted above has led to a shift in attention to a new experimental phase to evaluate its potential biological activity, also in eukaryotic cells. In particular, given the cosmetic interest in the finding of natural bioactive compounds, a cell line of immortalised human keratinocytes (HaCaT cells) was chosen as a reference system, and the biocompatibility of the crude extract of *F. proliferatum*, MBC, MFR and IAA with this type of cell was evaluated. As shown in Figure 9, the analysis of cell viability performed by MTT assay after 6, 24 and 48 h has highlighted that the crude extract was biocompatible with HaCaT cells, showing a slight reduction in cell viability only at the higher concentration and after 48 h, while MBC did not induce a significant toxic effect at higher concentrations tested until 24 h but toxic effects have been observed only after 48 h. Moreover, MFR resulted in slight toxicity at the doses and treatment times tested, and IAA showed significantly more toxic effects in a dose‐dependent manner at 48 h (Figure 9).

**FIGURE 10:**
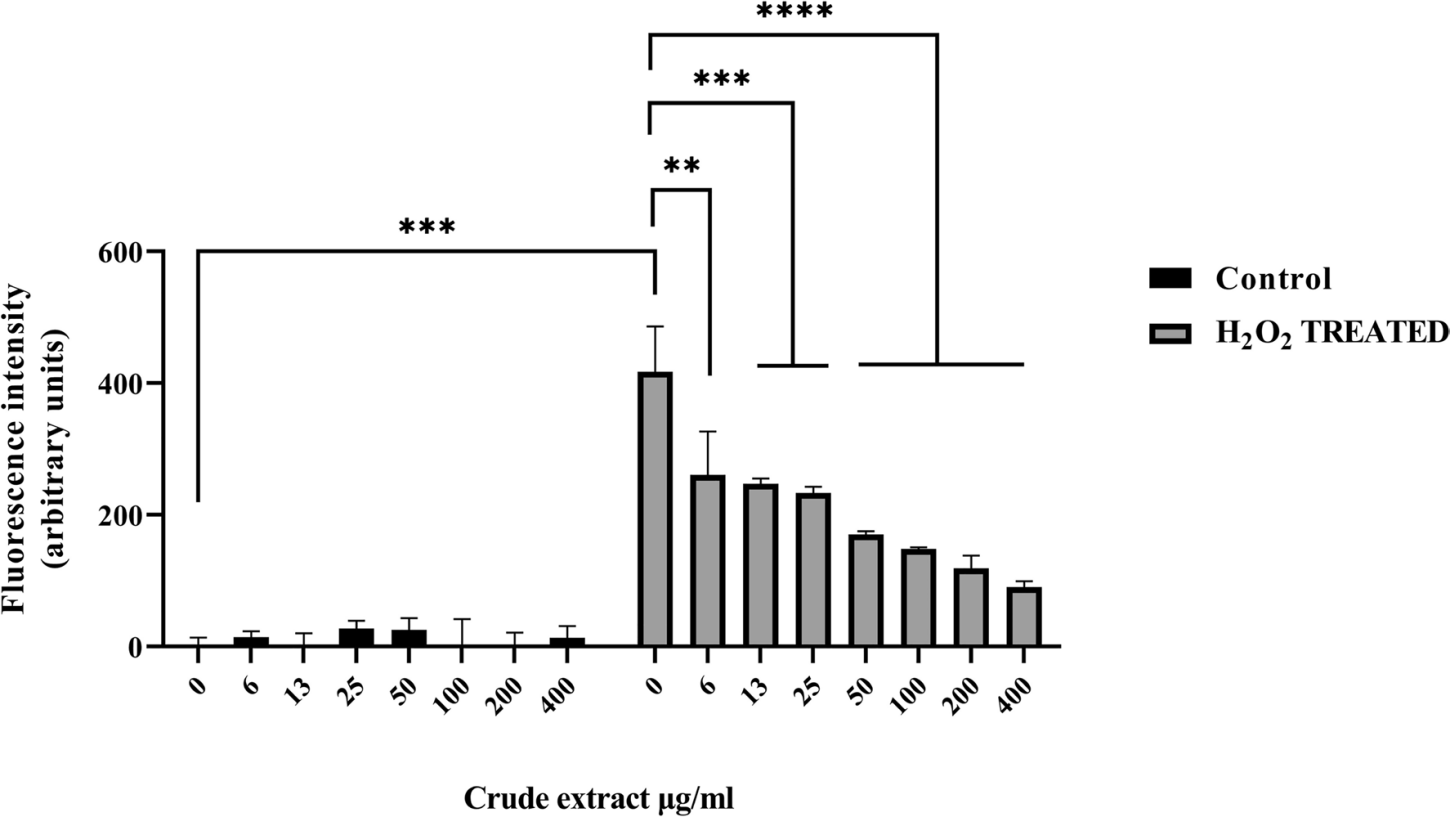
ROS release detection in HaCaT cells subjected to oxidative stress in the presence of increasing doses of crude extract. Values are the means ± SD of biological replicates (*p < 0.05, **p < 0.01, ***p < 0.001, ****p < 0.0001) compared to the respective controls (one‐way ANOVA, followed by Bonferroni's posttest). See Methods section for experimental details.

Once established that the crude extract resulted in the most biocompatible on HaCaT cells, a DCFH‐DA assay was carried out in order to evaluate its potential antioxidant activity. For this purpose, cells were treated with increasing amounts of crude extract (up to 400 μg mL^−1^) in absence or in presence of 100 μM of H_2_O_2_. As shown in Figure 10, the extract itself was not able to induce oxidative stress, while interestingly it was capable of significantly reducing in a dose‐dependent manner, the ROS production when cells are stimulated with H_2_O_2_.

## 4 Discussion

Nowadays, a major challenge in healthcare is represented by the rise of multidrug‐resistant pathogens. Such infections are difficult to treat and often require complex, costly and more toxic antibiotic regimens. Therefore, the search for novel, effective and affordable alternatives is crucial, and these potential solutions may lie within unexplored natural products. For this reason, in the present study, the crude extract and the secondary metabolites extracted from the de novo isolate *Fusarium proliferatum* strain 948,311 were analysed for their chemical composition, antioxidant potential, antimicrobial/antifungal activity and biocompatibility with human HaCaT cells. The pure compounds 9‐*O*‐methylbostrycoidin (MBC), 9‐*O*‐methylfusarubin (MFR) and 3‐indoleacetic acid (IAA), detected after purification of the crude organic extract with a higher extraction yield compared to other compounds, were selected for conducting the assays. The selection of the compounds showing a high extraction yield was functional to the potential industrial applicability of the single molecules, showing higher biological activity at lower concentrations compared to the crude extract.

### 4.1 Whole Genome Sequencing and Molecular Characteristics

Following the chemical characterisation of the fungus extracts and the preliminary molecular identification, using Sanger sequencing (targeting the ITS1‐ITS4 region amplifying the highly variable ITS1 and ITS2 sequences surrounding the 5.8S‐coding sequence), the Whole Genome Sequencing analysis was performed on the strain, allowing the de novo classification of the strain as *Fusarium proliferatum* strain FPI024. The classification allowed the confirmation of the morphology and the ITS sequencing. This Whole Genome Shotgun project has been deposited at DDBJ/ENA/GenBank under the accession JBJKOO010000000.

In order to confirm the capability of the strain to produce the isolated compounds, a comprehensive search analysis was conducted within the genome of *Fusarium proliferatum* for the genes involved in the synthesis of the MBC, MFR and IAA molecules. This analysis revealed the presence of the *fsr1*–*fsr6* gene cluster, which is responsible for the biosynthesis of fusarubin‐like metabolites (Studt et al. 2012). In addition, the fungal homologues of the bacterial IaaM and IaaH genes, which are responsible for the synthesis of the plant hormone IAA, were also identified. The existing body of literature on fusarubin is limited, with the biosynthesis of 9‐*O*‐methyl fusarubin being the only aspect that has been clearly delineated (Studt et al. 2012). The fusarubin gene cluster, present in all sequenced Fusaria genomes, contains six genes (*fsr1*–*fsr6*), of which *fsr1*–*fsr3* play essential roles in the biosynthesis of fusarubin‐like metabolites, such as MFR, and their aza537 anthraquinone derivatives, such as MBC, in *F. graminearum*. In addition to the polyketide synthase (PKS) FSR1 (PKS3, PGL1), the two enzymes necessary to produce the end products in these species are O‐methyltransferase (*fsr2*) and FAD‐binding monooxygenase (*fsr3*) (Pedersen et al. 2020). The plant hormone IAA can be biosynthesised from tryptophan via the intermediate indole‐3‐acetamide (IAM). The two genes, IaaM (encoding tryptophan monooxygenase) and IaaH (encoding indole‐3‐acetamide hydrolase) that constitute the IAM pathway, have been described in plant‐associated bacteria.

### 4.2 Purified Secondary Metabolites

The three compounds identified and purified from the crude extract reporting the highest yield were 9‐*O*‐methylbostrycoidin (MBC), 9‐*O*‐methylfusarubin (MFR) and 3‐indoleacetic acid (IAA) (Figure 4).

MBC is a polyketide metabolite, whose isolation and identification from multiple *Fusarium* species were previously reported. MBC belongs to the aza‐anthraquinone family and can be distinguished because of the characteristic deep‐red pigmentation (Gopalakrishnan et al. 2005; Steyn et al. 1979). MBC has also been previously isolated from the liquid cultures of the mangrove endophytic fungus *Aspergillus terreus* (No. GX7‐3B) (Deng et al. 2013) and from the endolichenic fungus *Corynespora* sp. BA‐10763 (Wijeratne et al. 2010).

MFR belongs to the class of naphthoquinones, a family of secondary metabolites produced by fungi of the *Fusarium* genus (Pessoa et al. 2017). MFR's structure consists of a modified naphthoquinone ring, which is responsible for its biological properties.

IAA is the most abundant auxin plant hormone, regulating various aspects of plant growth and development (Spaepen et al. 2007; Spaepen and Vanderleyden 2011). Both plants and microorganisms, including bacteria and fungi, are capable of producing IAA. Although some intermediates result differently, the IAA biosynthetic pathways in bacteria and plants are very similar. In particular, in plants and microbes, both tryptophan (Trp)‐dependent and Trp‐independent IAA biosynthetic pathways tend to co‐exist. Many environmental factors, including pH and temperature, are capable of influencing the IAA biosynthesis (Mano and Nemoto 2012; Spaepen et al. 2007). The extraction of IAA was also reported from the fungal derivative extract *F. proliferatum* AF‐04, isolated from the Chinese green onions (Toghueo 2019).

### 4.3 Antibacterial and Antifungal Activity of the Extract and Pure Metabolites

Over time, the *Fusarium* genus has proven its potential by producing numerous compounds approved for the treatment of serious diseases, including antimicrobial, anticancer, antimalarial, anti‐inflammatory and antioxidant therapies. These discoveries underscore the importance of further research to explore the vast chemical diversity found in these natural reservoirs.

Numerous studies in recent years reported the antimicrobial effect of the raw extract of various strains of *Fusarium proliferatum*. The raw extract of *Fusarium proliferatum* ZO‐L2‐4, an endophytic fungus isolated from *Zingiber officinale* Roscoe, demonstrated significant antimicrobial activity against *Staphylococcus aureus* , with a MIC value of 15.6 μg/mL (Ni Putu et al. 2025). Similarly, the raw ethyl acetate extract of *F. proliferatum* ACQR8, associated with *Cissus quadrangularis* L, exhibited significant broad‐spectrum antibacterial activity against both Gram‐positive and Gram‐negative bacteria. The lowest MIC values were observed against *Klebsiella pneumoniae* (with a 40 μg/mL concentration) and *Shigella boydii* (with a 46.67 μg/mL concentration), indicating remarkable efficacy against these pathogens. However, the compound exhibited elevated MICs against *Enterococcus faecalis* (with a 120 μg/mL concentration) and *Escherichia coli* (106.67 μg/mL), indicating reduced activity against these strains, while no zone of inhibition was detected for *Pseudomonas aeruginosa* . The same study reported a dose‐dependent inhibition of fungal colony diameter, except for *Candida albicans* , which remained insensitive to the extract at concentrations of up to 2 mg/mL in the culture medium (Singh et al. 2021).

According to the results obtained in the present study, following the evidence, through the Kirby‐Bauer disc diffusion assay, of a modest antibacterial and antifungal activity of the crude extract (1 mg) against *Staphylococcus aureus* ATCC 25923 (20.0 ± 0.2 mm), *Listeria monocytogenes* ATCC 19115, *Enterococcus hirae* ATCC 10541 and *Candida albicans* ATCC 16053, the broth microdilution method results allowed to demonstrate that the crude extract inhibited the growth of *S. aureus* in a dose‐dependent manner, reaching a 60% die‐off rate at a concentration of 100 μg mL^−1^. No activity was observed against Gram‐negative bacteria, such as *Escherichia coli* and *Pseudomonas aeruginosa* , suggesting that the extract does not cross the specific barriers of Gram‐negative bacteria, such as the outer membrane (Zgurskaya and Rybenkov 2020). The effective inhibitory concentrations in the reported studies result lower, compared to the present research: this could depend on the different extraction method, presumably allowing the recovery of different molecules in the raw extracts.

Despite these modest properties, the pure compounds isolated from the crude extract showed enhanced activities, demonstrating that the crude fraction potentially contains bioactive molecules holding a wide potential after the purification and concentration processes.

Analysing the results of the broth microdilution method assay, indeed, MBC highlighted a 100% die‐off rate of *S. aureus* ATCC 25923 at a 100 μg mL^−1^ concentration, > 90% rate at 50 μg mL^−1^ and a dose‐dependent antimicrobial activity at lower concentrations. An active concentration of 100 μg mL^−1^ of MBC was also able to inhibit the growth of *Listeria monocytogenes* ATCC 19115 and *Enterococcus hirae* ATCC 10541 (with 59.91% and 42.22% rates). The absence of activity against Gram‐negative bacteria and *Candida albicans* ATCC 16053 suggests that the molecular target of MBC might be specific for Gram‐positive bacteria, such as cell wall components or intracellular enzymes of the tested microorganisms. The reported outcomes are supported by previous studies reporting inhibition zones of 12–16 mm against *B. megaterium* and *S. aureus* (Khan et al. 2018).

In the present research, MFR reported a substantial inhibiting activity towards *S. aureus* and diverse Gram‐positive bacteria. A MFR concentration of 100 μg mL^−1^ caused the 98.01% die‐off of *S. aureus* ATCC 25923, with a MIC_50_ of 12.5 μg mL^−1^. MFR also showed an inhibitory effect on the growth of *Listeria monocytogenes* ATCC 19115, up to a 50% die‐off rate and of *Enterococcus hirae* ATCC 10541 up to a 60% die‐off rate. Literature data on fusarubin‐like metabolites extracted from other *Fusarium* species support the obtained results. MFR, indeed, showed bactericidal activity against some Gram‐positive bacteria, while it was inactive when tested against Gram‐negative bacteria (Baker et al. 1990). It was reported that MFR holds relevant antibacterial activity against *B. megaterium* and *S. aureus* , with inhibition zones ranging from 21 to 32 mm, indicating a broad spectrum of activity (Khan et al. 2018). Other analogues, such as the 3‐*O*‐methyl fusarubin and fusarubin isolates from *Fusarium solani* isolated from *Glycyrrhiza glabra* , showed antimicrobial activity against various bacterial strains such as *E. coli, S. pyogenes, Klebsiella* sp., *B* *. cereus* and *S. aureus* , beyond the inhibition of the H37Rv strain of *Mycobacterium tuberculosis* (Shah et al. 2017). The 3‐*O*‐methyl ether analogue of MFR, extracted from *F. proliferatum* AF‐04 isolated from the Chinese green onion, highlighted a selective and potent antibacterial activity against different microorganisms and pathogens, such as *B. megaterium, B* *. subtilis* , *C. perfringens, E. coli*, MRSA *and* RN4220, with a 50.0 μg mL^−1^ MIC against *B. megaterium* , comparable to ampicillin and erythromycin and stronger than streptomycin (Ahmed et al. 2023). In contrast, 3‐*O*‐methyl‐9‐*O*‐methyl fusarubin, isolated from *F. napiforme* collected from *Rhizophora mucronata* , exhibited moderate antibacterial activity against *S. aureus* and *P. aeruginosa* (6.3–12.5 μg mL^−1^ MICs), but showed no activity against *Aspergillus clavatus* or *Candida albicans* (Supratman et al. 2021). However, the MFR purified in this research only holds a maximum inhibition of 20% against *Candida albicans* ATCC 16053 at the highest concentration tested of 100 μg mL^−1^, allowing us to hypothesise that its antimicrobial effect on fungal species could be strain‐specific.

Given the obtained results, MBC and MFR could hold a potential industrial application as an antimicrobial substance, at a 50–100 μg mL^−1^.

Unlike MBC and MFR, IAA did not evidence antimicrobial activity at the tested concentrations. Nonetheless, the result is consistent with the structural nature of IAA, which is unlikely to have functional groups capable of interacting effectively with bacterial or fungal cellular components.

### 4.4 Biological Activity on HaCaT Cells

The crude extract resulted in the most bio‐compatible of the tested substances. HaCaT cells viability remained high even at higher concentrations and for long exposure times (48 h), highlighting a slight reduction only at the highest assayed doses. In the DCFH‐DA test, the extract did not induce intrinsic oxidative stress in HaCaT cells, demonstrating a safe profile. Notably, in the presence of hydrogen peroxide, it significantly reduced the production of reactive oxygen species (ROS) in a dose‐dependent way, suggesting a strong protective capacity against oxidative stress. Despite these modest properties, the pure compounds isolated from the crude extract subsequently showed enhanced activities, demonstrating that the crude fraction potentially contains bioactive molecules holding a wide potential after the purification and concentration processes.

In preliminary cytotoxicity tests performed, MBC did not induce a significant toxic effect up to 24 h at the higher concentrations tested, but toxic effects were evident at 48 h at higher doses. This behaviour might depend on the intracellular accumulation or specific interactions with long‐term metabolic processes. An analysis of the bibliography indicates that bostrycoidin showed remarkable inhibitory activity against α‐acetylcholinesterase (AChE) with a 6.71 μM IC_50_ value (Shweta et al. 2010), inhibited hepatic glucose production with an IC_50_ value of 30 μM and showed weak cytotoxicity against liver cells after incubation for 48 h (Shweta et al. 2010). MBC showed variable cytotoxic effect on vero cells, where it caused the death of almost 25% of the cells, indicating that the cell proliferation inhibitory activity of this compound would be useful in the antitumour treatment of renal cancer patients (Khan et al. 2018). MBC additionally showed significant free radical scavenging activity with 1.6, 12.4, 28.9 and 34.8 μg mL^−1^ IC_50_ values compared to BHA, Trolox and ascorbic acid as positive controls (Khan et al. 2018): compared to available studies, the compound isolated in this study did not show antioxidant activity using ORAC assay.

MFR showed a moderate toxicity towards HaCaT cells (immortalised human keratinocytes), with dose‐dependent effects observed after 48 h of treatment. Most interestingly, MFR results are less cytotoxic than the crude extract, MBC and 3‐indolacetic acid. Furthermore, the beneficial properties of MFR have been extensively studied in the literature: in particular, MFR showed significant activity against cancer cells, such as MCF‐7, with high selectivity (Vijitphan et al. 2019). Additionally, fusarubin isolated from *Fusarium solani* evidenced significant neuroprotective activity against glutamate‐mediated cell death in HT‐22 cells. In parallel, MFR exhibited selective cytotoxicity on Vero cells, causing 35% cell death, suggesting its potential as a cell proliferation inhibitor in oncological applications, particularly for the treatment of renal cancer.

IAA showed an interesting antioxidant activity, registering a maximum TEAC of 360.24 at 25 μg mL^−1^. Although IAA shows considerable antioxidant activity, the biocompatibility data indicate a dose‐dependent toxicity on HaCaT cells, with significant effects at 48 h. This toxicity limits the direct applicability of IAA, but its antioxidant profile could make it useful in formulations with systems that modulate its bioavailability.

In conclusion, the outcomes indicate that, although the crude extract has limited antimicrobial activity, the purified compounds have greater potential and could be exploited especially against harmful pathogens such as *S. aureus, L. monocytogenes and E. hirae*. Furthermore, the importance of performing additional studies to fully exploit the biotechnological potential of these compounds is confirmed by the safety profile and antioxidant properties of the crude extract and Indole‐3‐acetic acid. Ultimately, considering that marine fungi are still extremely underexplored, and the possible changes in their genomes and metabolomes in response to environmental adaptation are likewise unknown, the peculiar investigations on fungal assemblages could lead to the isolation of multiple novel compounds for diverse promising technological exploitation.

## Author Contributions

**Marco Masi** and **Federica Carraturo:** conceptualization (equal), data curation (equal), formal analysis (supporting), funding acquisition (equal), investigation (equal), methodology (equal), supervision (equal), validation (equal), visualization (equal), writing – original draft (equal), writing – review and editing (equal). **Marco Guida** and **Francesco Aliberti:** funding acquisition (equal), writing – review and editing (equal). **Antonio Nappo** and **Michela Salamone:** data curation (equal), formal analysis (supporting), writing – original draft (equal), writing – review and editing (equal). **Ilaria Di Nardo** and **Ilaria Di Nardo:** methodology (supporting), writing – original draft (supporting). **Alessio Cimmino, Andrea Bosso** and **Elio Pizzo:** formal analysis (supporting), methodology (supporting), writing – original draft (supporting). **Michele Sonnessa:** formal analysis (equal), methodology (equal), writing – original draft (equal), writing – review and editing (equal), software (supporting). **Maria Costantini** and **Valerio Zupo:** writing – review and editing (supporting).

## Conflicts of Interest

The authors declare no conflicts of interest.

## Supporting information


**Figure S1.** ^1^H NMR spectrum of 9‐*O*‐methyl bostrycoidin (MBC) (CDCl_3_, 400 MHz).
**Figure S2**. ^1^H NMR spectrum of 9‐*O*‐methylfusarubin (MFR) (CDCl_3_, 400 MHz).
**Figure S3**. ^1^H NMR spectrum of 3‐indoleacetic acid (MBC) (CDCl_3_, 400 MHz).
**Figure S4**. ESI MS spectrum of 9‐*O*‐methyl bostrycoidin (MBC), recorded in positive mode.
**Figure S5**. ESI MS spectrum of 9‐*O*‐methylfusarubin (MFR), recorded in positive mode.
**Figure S6**. ESI MS spectrum of 3‐indoleacetic acid (MBC), recorded in negative mode.

## Data Availability

The data that supports the findings of this study are available in the supplementary material of this article.
