# Supplementary material for: Biotechnological Potential of a Novel Strain of Fusarium proliferatum, a Terrestrial Fungus Adapted to Marine Environment
Source: Environ Microbiol Rep. 2025 Jul 4;17(4):e70143. doi: 10.1111/1758-2229.70143 (PMC12231200; doi:10.1111/1758-2229.70143)
Supplement: Supplementary file 1 — Figure S1. 1H NMR spectrum of 9‐O‐methyl bostrycoidin (MBC) (CDCl3, 400 MHz). Figure S2. 1H NMR spectrum of 9‐O‐methylfusarubin (MFR) (CDCl3, 400 MHz). Figure S3. 1H NMR spectrum of 3‐indoleacetic acid (MBC) (CDCl3, 400 MHz). Figure S4. ESI MS spectrum of 9‐O‐methyl bostrycoidin (MBC), recorded in positive mode. Figure S5. ESI MS spectrum of 9‐O‐methylfusarubin (MFR), recorded in positive mode. Figure S6. ESI MS spectrum of 3‐indoleacetic acid (MBC), recorded in negative mode. [file EMI4-17-e70143-s001.docx]

**SUPPORTING INFORMATION**

**Biotechnological potential of a novel strain of *Fusarium proliferatum,* a terrestrial fungus adapted to marine environment**

Antonio Nappo^1,2,#^, Michela Salamone^2,#^, Marco Masi^3,*^, Michela Morelli^2^, Martina Annunziata^1,2^, Michele Sonnessa^4^, Alessio Cimmino^3^, Andrea Bosso^2^, Rosanna Culurciello^2^, Ilaria Di Nardo^2^, Elio Pizzo^2^_,_ Maria Costantini^5^, Valerio Zupo^5^, Francesco Aliberti^2,6^, Marco Guida^2,6^, Federica Carraturo^2,6^

^1^ Department of Experimental Medicine, University of Campania “Luigi Vanvitelli”, Naples, Italy

^2^ Department of Biology, University of Naples Federico II, 80126, Naples, Italy

^3^ Department of Chemical Sciences, University of Naples Federico II, 80126 Naples, Italy

^4^ Bio-Fab Research Srl, Via Mario Beltrami 5, 00135 Rome, Italy

^5^ Department of Marine Biotechnology, Stazione Zoologica Anton Dohrn, Villa Comunale, 80121 Napoli, Italy

^6^ HoloBiotics srl, UNINA Spinoff, Via Cinthia 26, I-80126, Naples, Italy

^#^ These Authors equally contributed to this work.

* Correspondence: [marco.masi@unina.it](mailto:marco.masi@unina.it)

**Supporting information list**

**Figure S1**. ^1^H NMR spectrum of 9-*O*-methyl bostrycoidin (MBC) (CDCl_3_, 400 MHz).

**Figure S2**. ^1^H NMR spectrum of 9-*O*-methylfusarubin (MFR) (CDCl_3_, 400 MHz).

**Figure S3**. ^1^H NMR spectrum of 3-indoleacetic acid (MBC) (CDCl_3_, 400 MHz).

**Figure S4**. ESI MS spectrum of 9-*O*-methyl bostrycoidin (MBC), recorded in positive mode.

**Figure S5**. ESI MS spectrum of 9-*O*-methylfusarubin (MFR), recorded in positive mode.

**Figure S6**. ESI MS spectrum of 3-indoleacetic acid (MBC), recorded in negative mode.


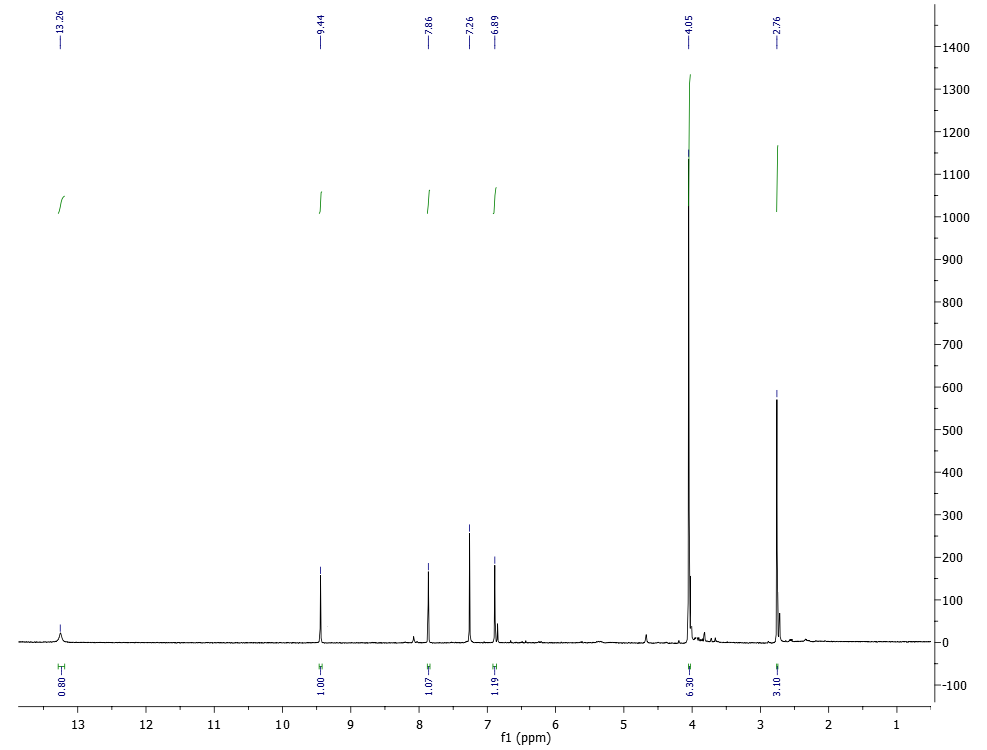


**Figure S1**. ^1^H NMR spectrum of 9-*O*-methyl bostrycoidin (MBC) (CDCl_3_, 400 MHz).


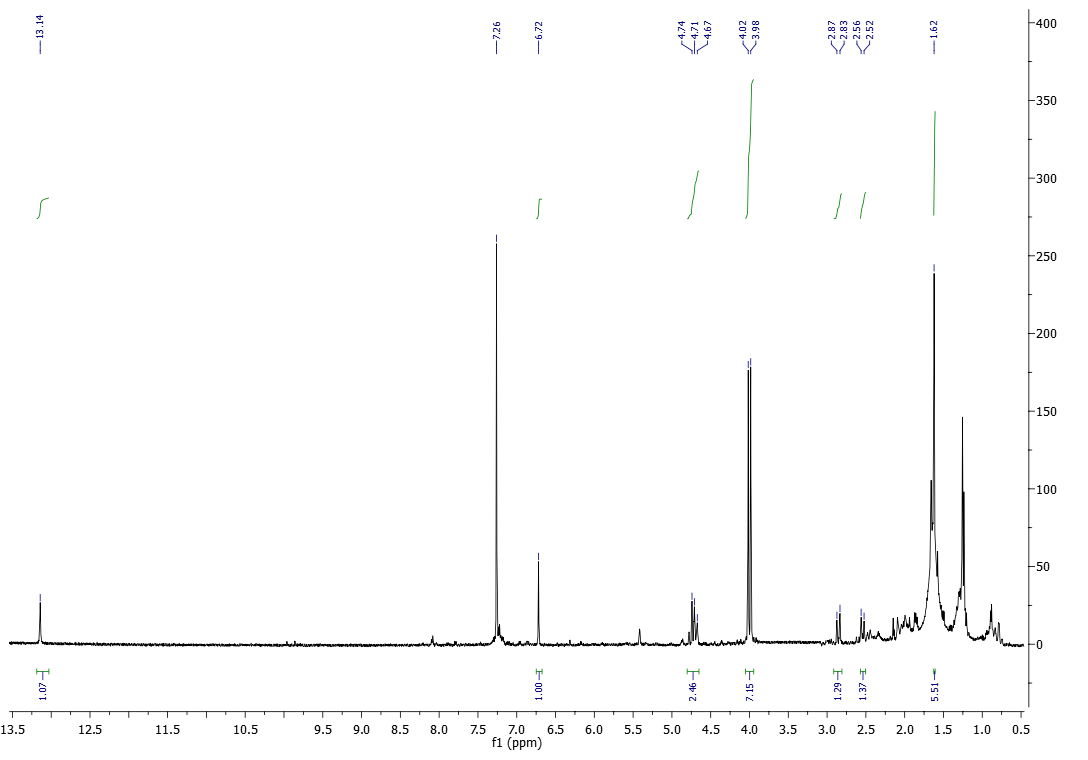


**Figure S2**. ^1^H NMR spectrum of 9-*O*-methylfusarubin (MFR) (CDCl_3_, 400 MHz).


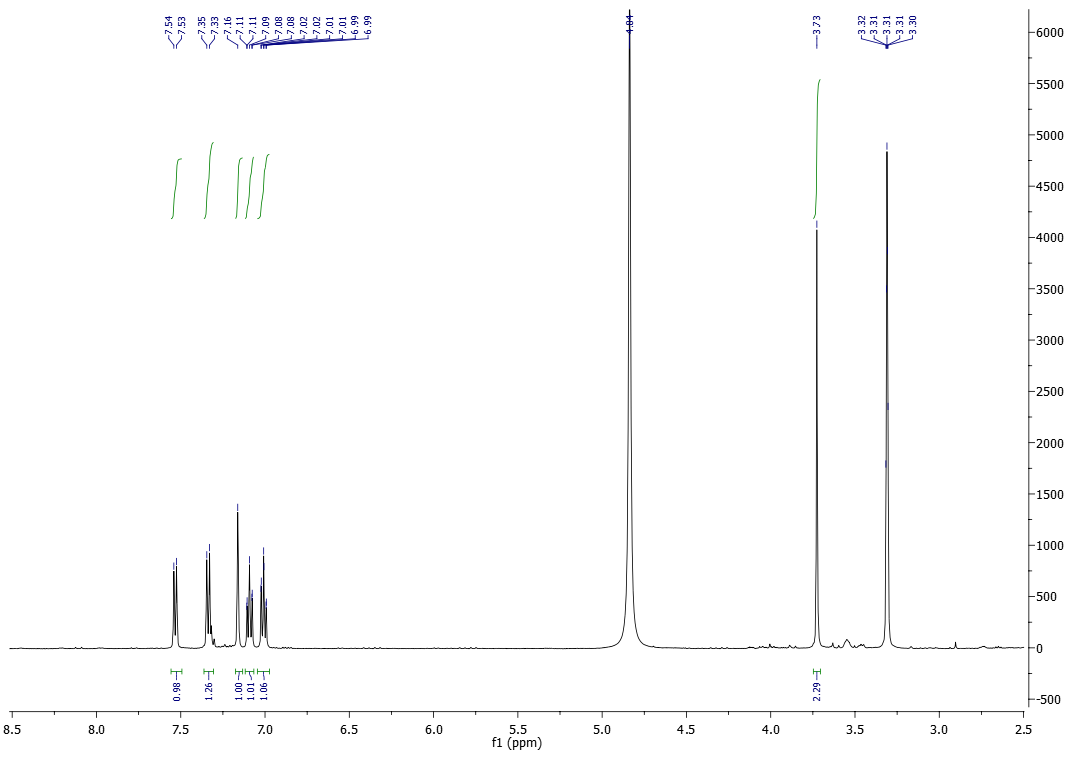


**Figure S3**. ^1^H NMR spectrum of 3-indoleacetic acid (MBC) (CDCl_3_, 400 MHz).

**
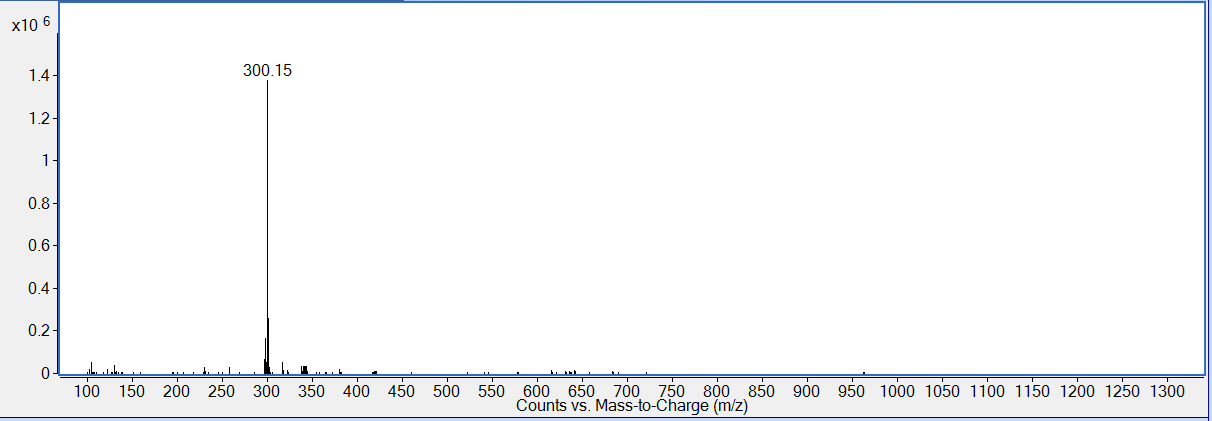
**

**Figure S4**. ESI MS spectrum of 9-*O*-methyl bostrycoidin (MBC), recorded in positive mode.

**
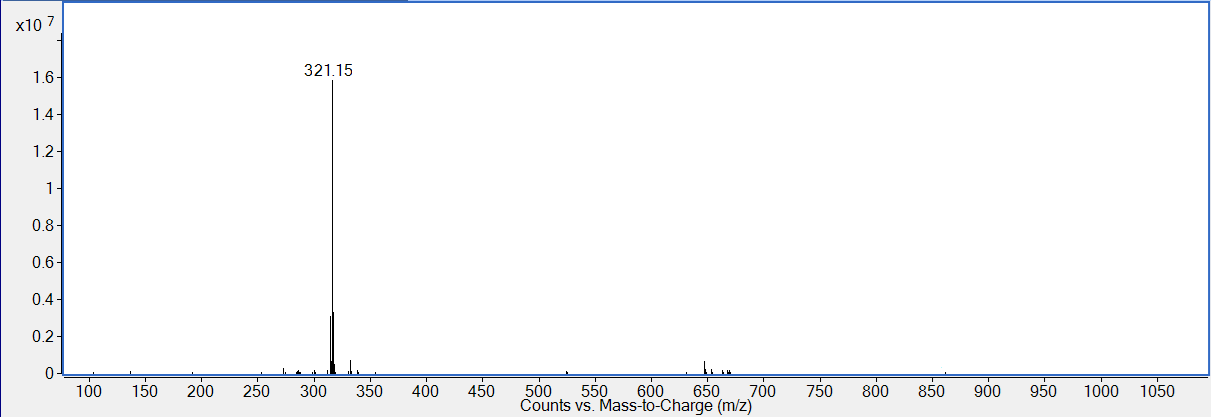
**

**Figure S5**. ESI MS spectrum of 9-*O*-methylfusarubin (MFR), recorded in positive mode.

**
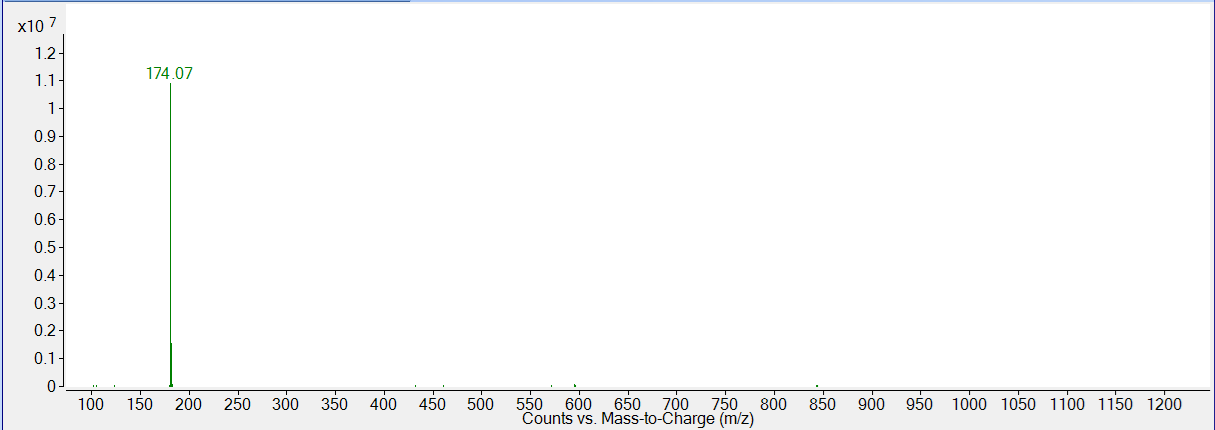
**

**Figure S6**. ESI MS spectrum of 3-indoleacetic acid (MBC), recorded in negative mode.
